# Detection of human, porcine and canine picornaviruses in municipal sewage sludge using pan-enterovirus amplicon-based long-read Illumina sequencing

**DOI:** 10.1080/22221751.2022.2071173

**Published:** 2022-05-23

**Authors:** Temitope O. C. Faleye, Erin M. Driver, Devin A. Bowes, Rochelle H. Holm, Daymond Talley, Ray Yeager, Aruni Bhatnagar, Ted Smith, Arvind Varsani, Rolf U. Halden, Matthew Scotch

**Affiliations:** a Biodesign Center for Environmental Health Engineering, Biodesign Institute, Arizona State University, Tempe, AZ, USA; b Christina Lee Brown Environment Institute, University of Louisville, Louisville, KY, USA; c Louisville/Jefferson County Metropolitan Sewer District, Morris Forman Water Quality Treatment Center, Louisville, KY, USA; d Biodesign Center for Fundamental and Applied Microbiomics, Center for Evolution and Medicine, School of Life Sciences, Arizona State University, Tempe, AZ USA; e College of Health Solutions, Arizona State University, Phoenix, AZ, USA

**Keywords:** Picornaviridae, wastewater-based epidemiological monitoring, high-throughput nucleotide sequencing, Kentucky, One Health

## Abstract

We describe the successful detection of human, porcine and canine picornaviruses (CanPV) in sewage sludge (at each stage of treatment) from Louisville, Kentucky, USA, using Pan-enterovirus amplicon-based long-read Illumina sequencing. Based on publicly available sequence data in GenBank, this is the first detection of CanPV in the USA and the first detection globally using wastewater-based epidemiology. Our findings also suggest there might be clusters of endemic porcine enterovirus (which have been shown capable of causing systemic infection in porcine) circulation in the USA that have not been sampled for around two decades. Our findings highlight the value of WBE coupled with amplicon based long-read Illumina sequencing for virus surveillance and demonstrates this approach can provide an avenue that supports a “One Health” model to virus surveillance. Finally, we describe a new CanPV assay targeting the capsid protein gene region that can be used globally, especially in resource limited settings for its detection and molecular epidemiology.

**To the Editor**: In situations where most pathogenic, human-infecting virus infections do not result in clinical manifestations, such as with Enteroviruses (EVs) [1], case-based surveillance (CBS) systems lack early detection capacity which is central for mitigating outbreaks before they result in significant morbidity and mortality. Considering most infected people shed viruses (or virus components such as nucleic acid) in large quantities in feces and consequently into wastewater, wastewater-based epidemiology (WBE) has consistently demonstrated capacity to function as an early warning system [2,3] and result in significant time and resource savings by facilitating surveillance of hundreds to thousands of people per sampling event.

We investigated the feasibility of using sludge from different stages of conventional wastewater treatment (primary sludge [PS], waste activated sludge [WAS] and dewatered sludge [centrifuged cake or CC]) for virus surveillance using EVs as a prototype virus. EVs are members of the genus *Enterovirus* (which has over 300 distinct types classified into 15 species) in the family *Picornaviridae*. EVs infect both humans and animals and in the USA are responsible for around 15 million human infections and tens of thousands of hospitalizations annually [4]. Though, over 90% of EV infected individuals are asymptomatic, all infected individuals excrete about 10^8^ virus particles/gram of feces (and consequently into wastewater) and shedding continues intermittently for weeks [1,5]. EVs are naked viruses with icosahedral symmetry that are very stable for elongated periods in the environment [1].

In June 2020, nine total sewage sludge samples [PS, WAS and CC] were collected (three per week), over three weeks (Figure 1a) from the Morris Forman Water Quality Treatment Center in Louisville, Kentucky, which serves a catchment with a population of ∼350,000 people. All samples were subjected to RNA extraction and complete EV capsid RT–PCR (Assay 1, Figure 1b,c) [6]. Subsequently, EV presence per sample was ascertained using assay 2 alongside Sanger sequencing (SS) (Figure 1b,c) [6]. This identified five samples as reliably containing EVs (Table S1). Three contained *Enterovirus Species G* (EV-G) members while each of the remaining two contained CVA11 (EV-C) and multiple peaks (suggestive of more than one EV type, Figure S1), respectively.

**Figure 1.**
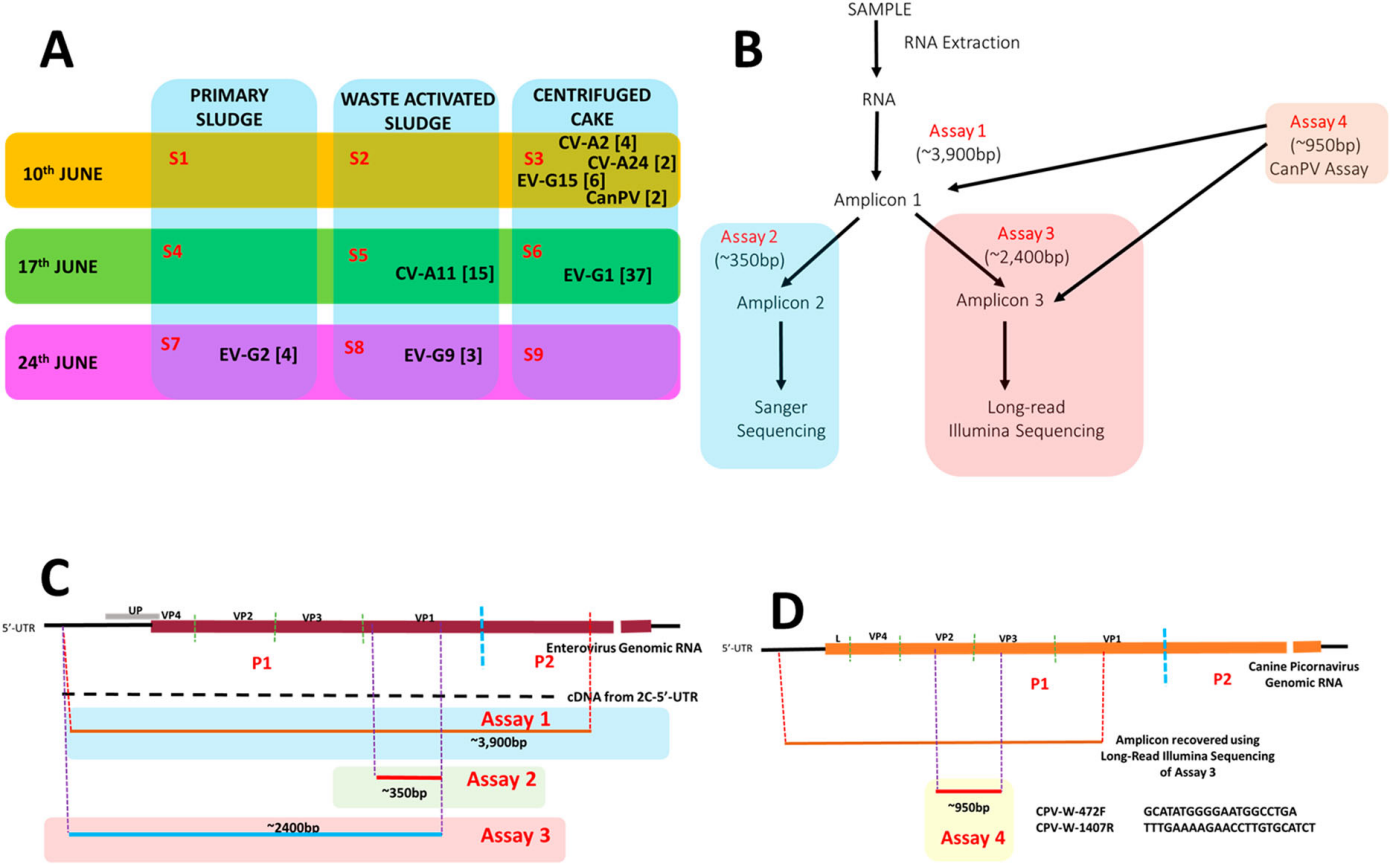
(A) Virus types detected in this study. S1-S9 refer to Samples 1-9. Numbers in bracket refer to the number of variants per virus type. (B) Schematic representation of the workflow used in this study, (C) EV genomic region amplified by Assays 1, 2 and 3, respectively (D) and CanPV genomic region recovered by long-read Illumina sequencing of amplicon from assay 3 and amplified by assay 4.

Assay 1 amplicons from these five confirmed EV positive samples were subjected to assay 3 and Long-read Illumina sequencing (LRIS). Seventy-three long-read contigs were recovered from the five EV positive samples (Table S2). Though more variants were recovered using LRIS, both SS and LRIS were congruent with respect to the EV types detected in four (samples 5, 6, 7, and 8) of the five samples (Tables S1 and S2). SS showed multiple peaks in the fifth sample (Sample 3, Tables S1) while LRIS delineated the different virus types (Figure 1a and Table S2) and variants present in the sample. LRIS also showed the presence of two canine picornavirus variants in the sample (Table S2 and S3).

Since unlike for EV-A and EV-C, the enterovirus genotyping tool (EGT) [7] does not resolve EV-G species members into types (Table S3), we used a combination of phylogenetic and pairwise identity analysis to type the EV-Gs, and found them belonging to genotypes 1, 2, 9 and 15 (Figure S2). Pairwise identity analysis showed that the EV-Gs detected in this study were ∼20% divergent (Table S4 and Figure S3) from the most similar sequence in GenBank (even those detected in California, USA in 2018 [8] [Figure S3]) suggesting these might have circulated undescribed for around two decades (at an evolutionary rate of 1 × 10^−2^ substitutions per site per year [i.e. ∼1% divergence per year] [9]). A similar observation was made for the EV-Cs (CVA11 and CVA24) which were 16% to 20% divergent (Table S4) from the most similar sequence in GenBank. The EV-A (CVA2) was different in that the most similar sequence in GenBank was ∼3% divergent (MT641397; found in a respiratory specimen in the UK in 2018) (Table S4).

Phylogenetic analysis of the two CanPV contigs (Figure S4) showed that they belong to a group of unclassified canine picornaviruses that (based on publicly available sequence data in GenBank) have not been previously described in the USA. They have however been described in dogs in the United Arab Emirates (UAE), China and Hong Kong for over a decade (2008 to 2019) [10–12] and more recently in Foxes in Australia [13] but <10 sequences are publicly available in GenBank as of the 23rd of March 2022. Since CanPV detection as described above was serendipitous, to confirm it was truly present in our sample, we designed assay 4 (Figure 1d) and subjected both assays 1 and 3 amplicon from sample 3 to the assay (assay 4, Figure 1b). We succeeded in amplifying the ∼950 bp amplicon from both (Figure S5) and Sanger sequencing confirmed that CanPV was, in fact, present. This suggests that CanPV amplification occurred first in assay 1. In fact, we have subsequently recovered multiple variants of CanPV (with the same contig size) in an independent study using samples from another state in the USA (unpublished data) in which we sequenced products from assay 1 using Illumina technology. This confirmed that near complete CanPV capsid region could be amplified using assay 1 and showed divergence bordering ∼20% between CanPV capsid variants circulating in the USA between 2019 and 2021 (unpublished data).

Our findings show that sludge from different stages (PS, WAS and CC) of conventional wastewater treatment can be used for virus surveillance. We recovered porcine (EV-G), canine (CanPV) and human (EV-A and EV-C) picornaviruses demonstrating this approach provides an avenue that facilitates surveillance of both human viruses and animal viruses and a *One-Health* framework [14]. In addition, our findings document the existence of both human and animal virus (with potential to cause significant morbidity and mortality) lineages that have been circulating in the USA for around two decades undetected. Finally, we document (based on publicly available sequence data in GenBank) the first detection of CanPV in the USA and the first detection globally using wastewater-based epidemiology. Considering the dearth of information on CanPV (with <10 sequences publicly available in GenBank as of 23rd March 2022) we describe a new CanPV assay (Figure 1d) targeting the capsid protein gene region that can be used for CanPV detection and molecular epidemiology globally, especially in resource limited settings and thereby facilitate our understanding of its global dynamics.

## Supplementary Material

Supplemental Material

## Data Availability

Sequences generated from this study are available in NCBI GenBank under accession numbers OK554433 – OK554505 and OM782676.
