# Supplementary material for: Detection of human, porcine and canine picornaviruses in municipal sewage sludge using pan-enterovirus amplicon-based long-read Illumina sequencing
Source: Emerg Microbes Infect. 2022 May 23;11(1):1339–42. doi: 10.1080/22221751.2022.2071173 (PMC9132413; doi:10.1080/22221751.2022.2071173)
Supplement: Supplemental Material [file TEMI_A_2071173_SM2050.docx]

**SUPPLEMENTARY MATERIALS**

**Title:** Detection of Human, Porcine and Canine Picornaviruses in Municipal Sewage Sludge Using Pan-Enterovirus Amplicon-based Long-read Illumina Sequencing

**Authors:** Temitope O.C. Faleye^a^, Erin M. Driver^a^, Devin A. Bowes^a^, Rochelle H. Holm^b^, Daymond Talley^c^, Ray Yeager^b^, Aruni Bhatnagar^b^, Ted Smith^b^, Arvind Varsani^d^, Rolf U Halden^a^ and Matthew Scotch^a,e^ *

^a^Biodesign Center for Environmental Health Engineering, Biodesign Institute, Arizona State University, Tempe, AZ USA; ^b^Christina Lee Brown Environment Institute, University of Louisville, Louisville, KY USA; ^c^Louisville/Jefferson County Metropolitan Sewer District, Morris Forman Water Quality Treatment Center, Louisville, KY USA; ^d^Biodesign Center for Fundamental and Applied Microbiomics, Center for Evolution and Medicine, School of Life Sciences, Arizona State University, Tempe, AZ USA; ^e^College of Health Solutions, Arizona State University, Phoenix, AZ USA.

*Correspondence: matthew.scotch@asu.edu

**APPENDIX A**: **SUPPLEMENTARY METHODS**

1.1. Sample Collection

We collected and analyzed sewage sludge from the Morris Forman Water Quality Treatment Center (wastewater treatment plant [WWTP]) in Louisville, Kentucky, USA in June 2020. The WWTP sampled serves a catchment with a population of ~350,000 people. Three samples [PS, WAS and CC] were collected per day on the 10^th^, 17^th^ and 24^th^ of June. After collection, the samples were shipped overnight on ice to the Biodesign Institute, Arizona State University, Tempe, Arizona for processing.

1.2. Sample processing, RNA extraction and RT-PCR

We aliquoted 2.5 mL of each sample into a 50 mL centrifuge tube containing 1 g of plastic beads and 7.5 mL of nuclease free water. The mixture was vortexed for 20 minutes and afterwards centrifuged at 4,000 rpm for 20 minutes. The supernatant was collected and stored in 1 mL aliquots at -80^o^C.

Precisely, 280 µL (140 µL x 2) of each sample was subjected to RNA extraction using the QiaAmp RNA extraction kit (Qiagen, Hilden, Germany) following manufacturer’s recommendation. Subsequently, the RNA extract was subjected to the EV detection workflow described in Faleye *et al.* [1] with a slight modification (Figure S1). Specifically, 5 µL of RNA extract was subjected to the one-step RT-PCR assay (assay 1, Figure S1) described in [2]. The amplicon product of the assay was then used as template in two independent second round PCR assays yielding ~350 bp (assay 2, Figure S1) and ~2,400 bp (assay 3, Figure S1) amplicons, respectively as previously described [1]. Assay 2 and 3 amplify VP1 and 5’-UTR to VP1 regions of the EV genome respectively (Figure S2). Assay 2 was used to cost effectively streamline samples subjected to assay 3 and consequently, long-read Illumina sequencing. Please note that all sequencing reactions described here were outsourced to commercial facilities. Hence the Sanger and long-read Illumina sequencing results were also used as controls for each other.

The amplicon product of assays 1 and 3 were independently used as template for assay 4 (Figure S1) which amplifies an ~ 950bp region of the CanPV genome (Figure S3) spanning VP2-VP3 (nucleotide 1089 to 2067 relative to JN831356). Specifically, 2uL of each was added independently to a PCR master mix containing 12.5uL of GoTaq green, 0.25uL (100uM) each of forward and reverse primers and 10uL of PCR-grade water. Amplification was done in a Bio-Rad C1000 thermal cycler as follows; 94^o^C for 2 minutes, 35 cycles of 94^o^C for 15 seconds, 55^o^C for 30 seconds and 60^o^C for 60 seconds. This was followed by 68^o^C for 5 minutes and held at 4^o^C until stopped. The amplicons (~350 bp, ~950 bp and ~2400 bp) were resolved on 1% or 2% gels stained with GelRed (Biotium Inc., Fremont, California, USA).

1.3. Sequencing

The ~350 bp and ~ 950bp amplicons were Sanger sequenced at the Genomics Core at Arizona State University while the ~2,400 bp amplicon was indexed, normalized and pooled using Loop Genomics long-read sequencing kit (San Jose, California, USA) as recommended by the manufacturer. The pooled product was then shipped to Loop Genomics where library preparation and sequencing was done using their long-read sequencing technology on a HiSeq 4000 Illumina sequencer (2 × 150 paired-end). Assembly of short reads to long reads was also done using Loop Genomics pipeline [3] and Spades [4].

1.4. Genotyping

The EV contigs were identified using the enterovirus genotyping tool (EGT) version 1.0 [5] and duplicate contigs were removed using the ‘*find duplicates*’ tool in Geneious Prime v2020.1.2 (Biomatters Ltd., Auckland, New Zealand). For the EVs, a local database was created using sequences recovered in this study and similar sequences from GenBank [6]. Specifically, the EV contigs recovered in this study were subjected to a BLASTn [7] search of the GenBank database, the top 100 hits of each were recovered, pooled and duplicate sequences removed. Multiple sequence alignments were done using ClustalW in MEGAX [8] and Maximum-Likelihood (ML) trees were generated in MEGAX using 1,000 bootstrap replicates. Pairwise Identity analysis was performed using SDT v1.2. [9].

The CanPV contigs were identified using both the EGT and BLASTn search of the GenBank database. Subsequently, a local database was created as described above for EVs and subsequently *in silico* translated using the *orfFinder* web server; a National Center for Biotechnology Information (NCBI) resource [10]. The *in silico* translated database was then used to make ML phylogenetic trees as described above. Sequences generated in this study have been deposited in GenBank under the accession numbers OK554433 – OK554505 and OM782676.

**References**

1. Faleye TOC, Bowes DA, Driver EM, et al. Wastewater-Based Epidemiology and Long-Read Sequencing to Identify Enterovirus Circulation in Three Municipalities in Maricopa County, Arizona, Southwest United States between June and October 2020. Viruses. 2021; 13(9):1803. Available from: https://ncbi.nlm.nih.gov/pubmed/ 34578384.
2. Majumdar M, Martin J. Detection by Direct Next Generation Sequencing Analysis of Emerging Enterovirus D68 and C109 Strains in an Environmental Sample From Scotland. Front Microbiol. 2018;9:1956. Available from: https://ncbi.nlm.nih.gov/pubmed/30186268.
3. Callahan BJ, Grinevich D, Thakur S, et al. Ultra-accurate microbial amplicon sequencing with synthetic long reads. Microbiome. 2021;5;9(1):130. Available from: https://ncbi.nlm.nih.gov/pubmed/34090540.
4. Bankevich A, Nurk S, Antipov D, et al. SPAdes: a new genome assembly algorithm and its applications to single-cell sequencing. J Comput Biol. 2012;19(5):455-77. Available from: https://ncbi.nlm.nih.gov/pubmed/22506599.
5. Kroneman A, Vennema H, Deforche K, et al. An automated genotyping tool for enteroviruses and noroviruses. J Clin Virol. 2011;51(2):121-5. Available from: https://ncbi.nlm.nih.gov/pubmed/ 21514213.
6. Sayers EW, Cavanaugh M, Clark K, et al. GenBank. Nucleic Acids Res. 2021;49(D1):D92-D96. Available from: https://ncbi.nlm.nih.gov/pubmed/ 33196830.
7. Altschul SF, Gish W, Miller W, et al. Basic local alignment search tool. J Mol Biol. 1990;215(3):403-10. Available from: https://ncbi.nlm.nih.gov/pubmed/2231712.
8. Kumar S, Stecher G, Li M, et al. MEGA X: Molecular Evolutionary Genetics Analysis across Computing Platforms. Mol Biol Evol. 2018;35(6):1547-1549. Available from: https://ncbi.nlm.nih.gov/pubmed/ 29722887.
9. Muhire BM, Varsani A, Martin DP. SDT: a virus classification tool based on pairwise sequence alignment and identity calculation. PLoS One. 2014;9(9):e108277. Available from: https://ncbi.nlm.nih.gov/pubmed/ 25259891.
10. Sayers EW, Barrett T, Benson DA, et al. Database resources of the National Center for Biotechnology Information. Nucleic Acids Res. 2011;39(Database issue):D38-51. Available from: https://ncbi.nlm.nih.gov/pubmed/ 21097890.

**APPENDIX B**: **SUPPLEMENTARY FIGURES**


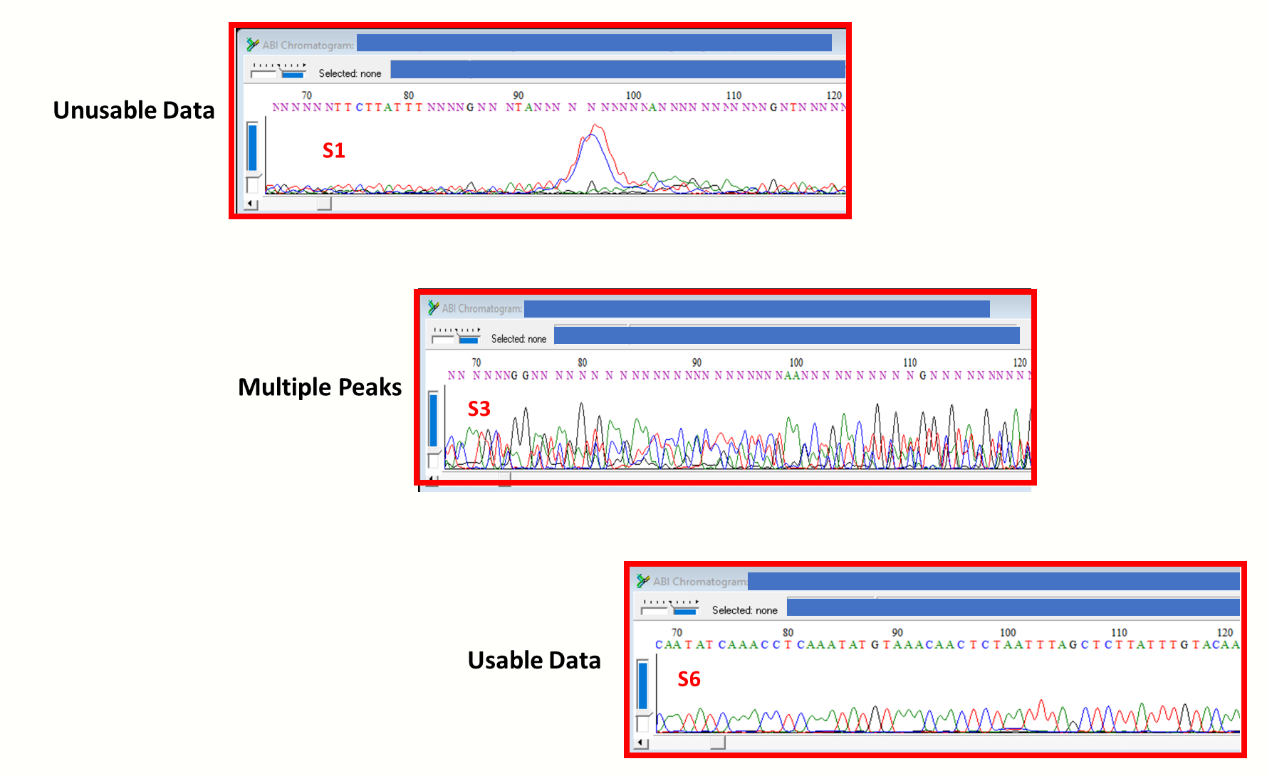


**Figure S1**: Samples of Sanger sequencing chromatogram data showing usable and unusable data and multiple peaks.


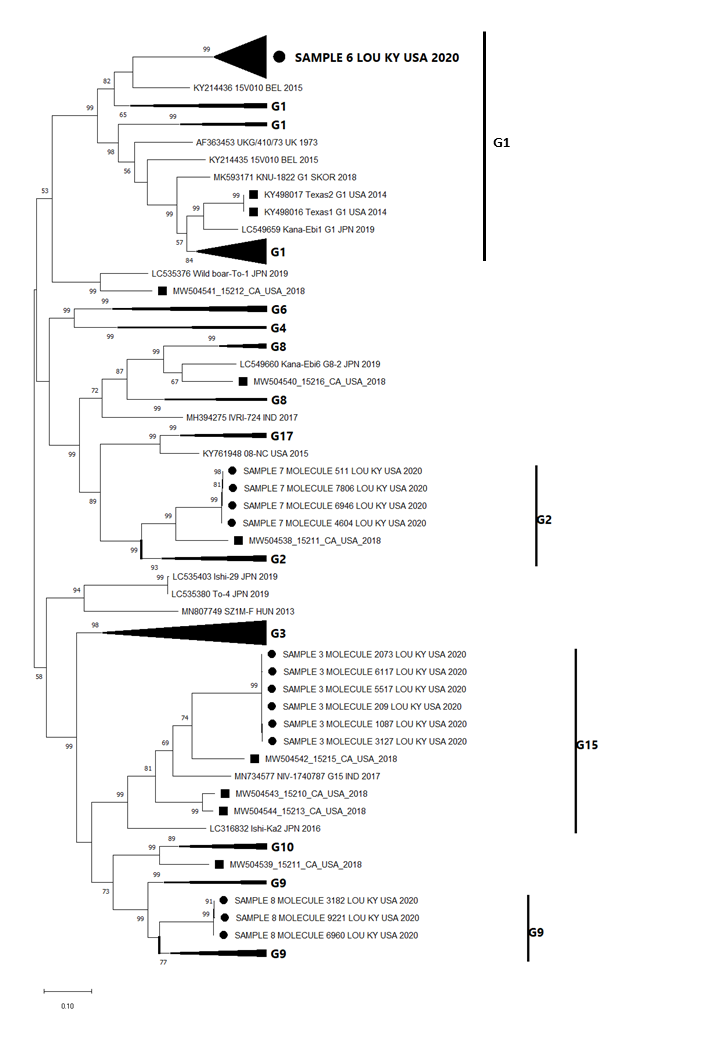


**Figure S2.** Identification of EVG contigs recovered in this study using the 2,400 bp contigs. Sequences described in this study and those previously described in the USA are labelled with black circle and square, respectively. Types bordered with black vertical lines (G1, G2, G9 and G15) connote types detected in this study. Bootstrap values are shown if >50%. Collapsed taxa denote all sequences therein belong to the same EVG type.


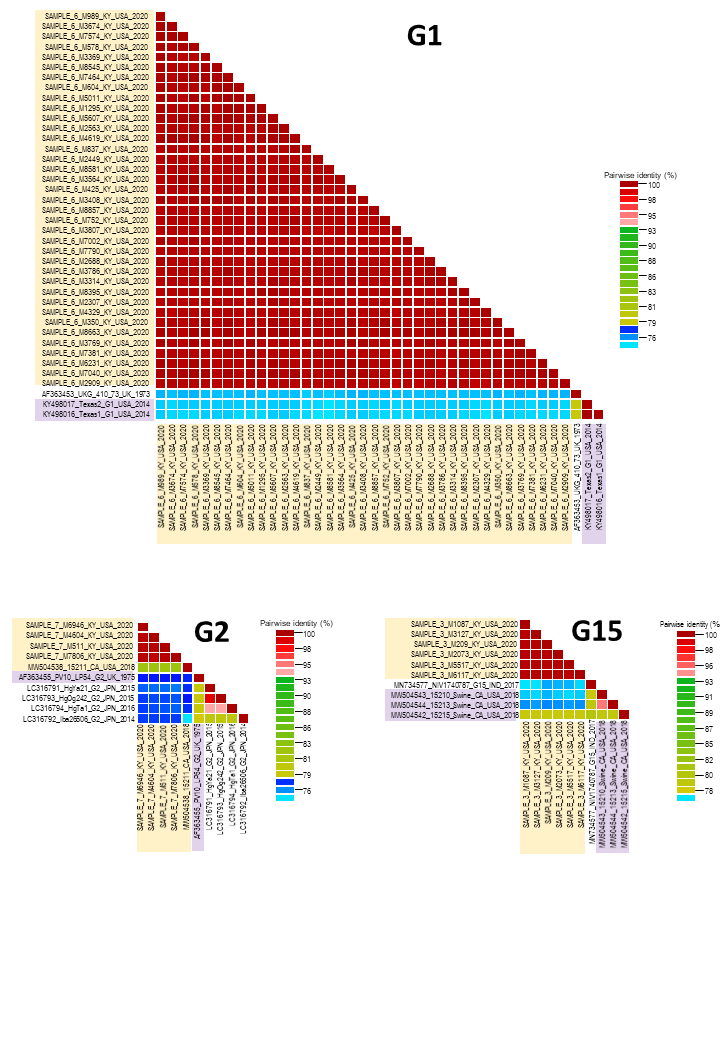


**Figure S3.** Pairwise identity analysis of EVG types found in this study for which sequences have been previously described in the USA. Sequences described in this study and those previously described in the USA are highlighted with yellow and purple, respectively.


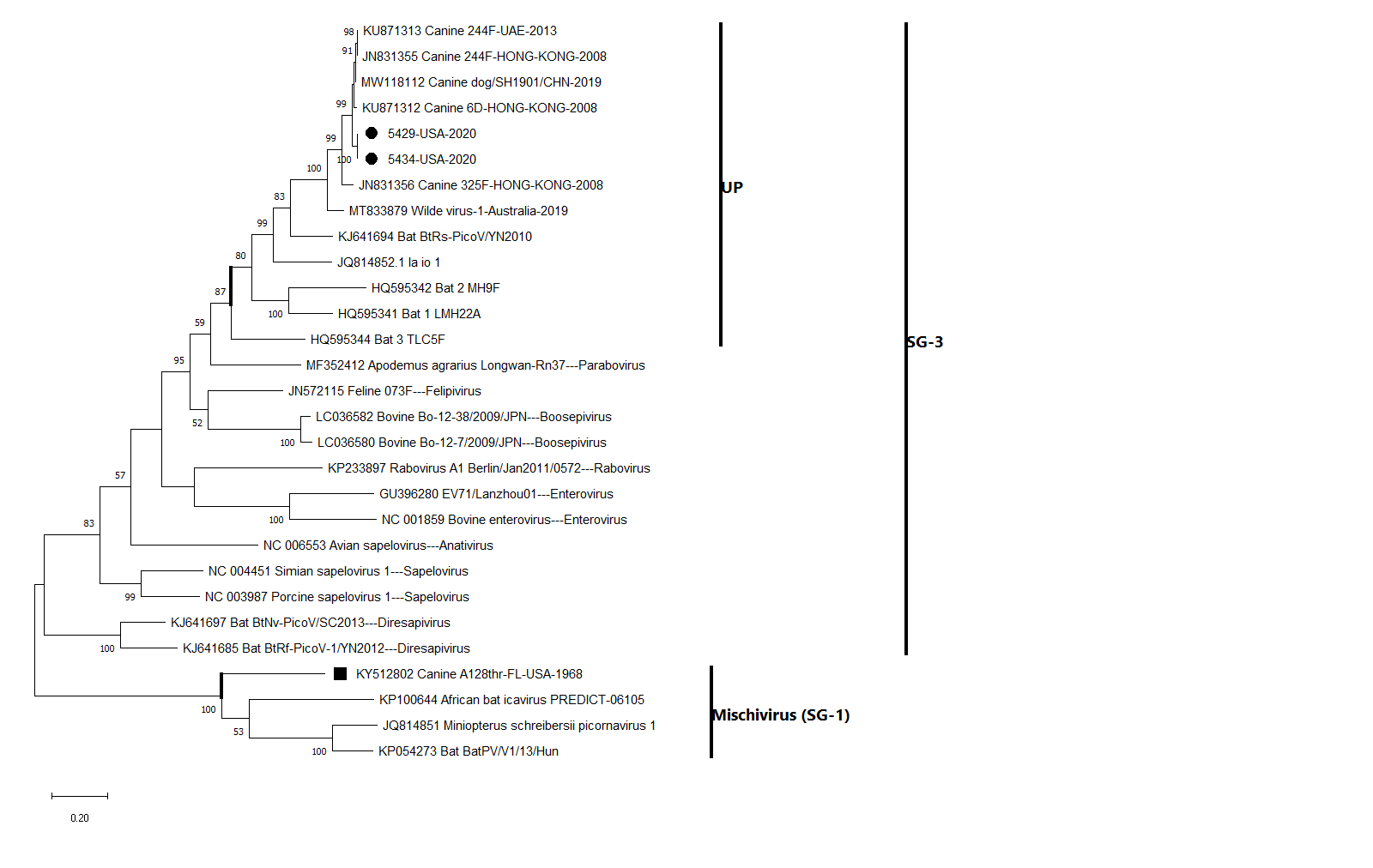


**Figure S4.** Phylogenetic tree for identification of canine picornavirus contigs recovered in this study. Sequences described in this study and those previously described in the USA are labelled with black circle and square, respectively. Bootstrap values are shown if >50%. Abbreviations: SG - Supergroup; UP - Unclassified Picornaviruses.


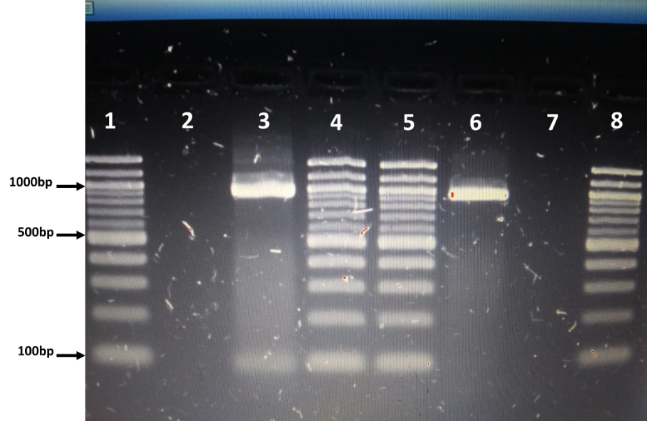


**Figure S5**: Assay 4 gel electrophoresis result. Lanes 1, 4, 5 and 8 contain 100bp ladder. Lanes 2 and 7 are empty. Lanes 3 and 6 show bands suggestive of successful CanPV detection using assay 4. Band in lane 3 was recovered by subjecting amplicon from assay 1 to assay 4. Band in lane 6 was recovered by subjecting amplicon from assay 3 to assay 4.

**Appendix C. SUPPLEMENTARY TABLES**

**Table S1.** Sanger sequencing results for EV. S-ID means sample identification number.

| **S-ID** | **Collection Date** | **Sludge type** | **EV-Screen** | **EV-type** |
| --- | --- | --- | --- | --- |
| 1 | 10^th^ June 2020 | Primary | Positive | Poor data |
| 2 | 10^th^ June 2020 | Waste Activated | Negative | NA |
| 3 | 10^th^ June 2020 | Centrifuged Cake | Positive | MP |
| 4 | 17^th^ June 2020 | Primary | Negative | NA |
| 5 | 17^th^ June 2020 | Waste Activated | Positive | CVA11 |
| 6 | 17^th^ June 2020 | Centrifuged Cake | Positive | EV-G |
| 7 | 24^th^ June 2020 | Primary | Positive | EV-G |
| 8 | 24^th^ June 2020 | Waste Activated | Positive | EV-G |
| 9 | 24^th^ June 2020 | Centrifuged Cake | Positive | Poor data |

**Table S2.** Illumina sequencing results for EV. S-ID means sample identification number.

| **S-ID** | **Short Reads** | **Assembled long Reads** | **Virus Long-reads** | **Enterovirus Long-reads** | **Virus-type (N)** | **Total Contigs** | **Short Reads Range** | **Coverage** |
| --- | --- | --- | --- | --- | --- | --- | --- | --- |
| 3 | 2,898,558 | 6,610 | 3,483 | 2,393 | CVA24 (2)/CVA2 (4)/EVG (6)/CP (2) | 14 | 1666 – 402 | 208x – 31x |
| 5 | 1,175,369 | 1,667 | 1,233 | 1,233 | CVA11 (15) | 15 | 1088 – 250 | 136x – 31x |
| 6 | 3,035,478 | 9,002 | 771 | 771 | EVG (37) | 37 | 1880 – 276 | 235x – 34x |
| 7 | 3,336,677 | 8,512 | 866 | 307 | EVG (4) | 4 | 1698 – 340 | 212x – 42x |
| 8 | 3,137,213 | 9,427 | 164 | 130 | EVG (3) | 3 | 1882 – 570 | 235x – 71x |
|  | **13,583,295** | **35,218** | **6,517** | **4,834** |  | **73** |  |  |

**Table S3.** Enterovirus Genotyping tool (EGT) virus identification results. Abbreviations: NA = Not Applicable; EV = Enterovirus, CanPV = Canine Picornavirus, CV = Coxsackievirus, VP = Virus protein.

| **Amplicon-Unique-ID** | **Begin** | **End** | **RefSeq** | **BLAST result** | **BLAST score** | **Type** | **Type support** | **VP1 type** | **VP1 type support** |
| --- | --- | --- | --- | --- | --- | --- | --- | --- | --- |
| S3_M1087 | 594 | 2935 | AF363455 | EVG | 69.41 | NA | NA | NA | NA |
| S3_M1819 | 537 | 2931 | NC_001612 | EVA | 80.91 | CV-A2 | 100 | CV-A2 | 100 |
| S3_M1933 | 528 | 2992 | NC_002058 | EVC | 79.46 | CV-A24 | 100 | CV-A24 | 94 |
| S3_M2073 | 594 | 2935 | AF363455 | EVG | 69.54 | NA | NA | NA | NA |
| S3_M 209 | 594 | 2935 | AF363455 | EVG | 69.54 | NA | NA | NA | NA |
| S3_M2103 | 528 | 2992 | NC_002058 | EVC | 79.38 | CV-A24 | 100 | CV-A24 | 95 |
| S3_M3127 | 594 | 2935 | AF363455 | EVG | 69.58 | NA | NA | NA | NA |
| S3_M3761 | 537 | 2931 | NC_001612 | EVA | 80.87 | CV-A2 | 100 | CV-A2 | 100 |
| S3_M4055 | 537 | 2931 | NC_001612 | EVA | 80.78 | CV-A2 | 100 | CV-A2 | 100 |
| S3_M5429 | 557 | 2879 | JN831356 | CanPV | 79.93 | NA | NA | NA | NA |
| S3_M5434 | 557 | 3030 | JN831356 | CanPV | 79.89 | NA | NA | NA | NA |
| S3_M5517 | 594 | 2935 | AF363455 | EVG | 69.50 | NA | NA | NA | NA |
| S3_M5564 | 537 | 2931 | NC_001612 | EVA | 80.87 | CV-A2 | 100 | CV-A2 | 100 |
| S3_M6117 | 594 | 2935 | AF363455 | EVG | 69.50 | NA | NA | NA | NA |
| S5_M1027 | 528 | 2989 | NC_002058 | EVC | 79.16 | CV-A11 | 100 | CV-A11 | 100 |
| S5_M1172 | 528 | 2989 | NC_002058 | EVC | 79.24 | CV-A11 | 100 | CV-A11 | 100 |
| S5_M1250 | 528 | 2989 | NC_002058 | EVC | 79.04 | CV-A11 | 100 | CV-A11 | 100 |
| S5_M1441 | 528 | 2989 | NC_002058 | EVC | 79.08 | CV-A11 | 100 | CV-A11 | 100 |
| S5_M1500 | 528 | 2989 | NC_002058 | EVC | 81.30 | CV-A11 | 100 | CV-A11 | 100 |
| S5_M1560 | 528 | 2989 | NC_002058 | EVC | 81.52 | CV-A11 | 100 | CV-A11 | 100 |
| S5_M1651 | 528 | 2989 | NC_002058 | EVC | 79.08 | CV-A11 | 100 | CV-A11 | 100 |
| S5_M167 | 528 | 2989 | NC_002058 | EVC | 81.65 | CV-A11 | 100 | CV-A11 | 100 |
| S5_M207 | 528 | 2989 | NC_002058 | EVC | 79.00 | CV-A11 | 100 | CV-A11 | 100 |
| S5_M237 | 528 | 2989 | NC_002058 | EVC | 79.08 | CV-A11 | 100 | CV-A11 | 100 |
| S5_M238 | 528 | 2988 | NC_002058 | EVC | 81.47 | CV-A11 | 100 | CV-A11 | 100 |
| S5_M248 | 528 | 2989 | NC_002058 | EVC | 79.12 | CV-A11 | 100 | CV-A11 | 100 |
| S5_M307 | 528 | 2989 | NC_002058 | EVC | 81.52 | CV-A11 | 100 | CV-A11 | 100 |
| S5_M352 | 528 | 2989 | NC_002058 | EVC | 79.16 | CV-A11 | 100 | CV-A11 | 100 |
| S5_M447 | 528 | 2989 | NC_002058 | EVC | 79.04 | CV-A11 | 100 | CV-A11 | 100 |
| S5_M476 | 528 | 2989 | NC_002058 | EVC | 79.12 | CV-A11 | 100 | CV-A11 | 100 |
| S5_M554 | 528 | 2989 | NC_002058 | EVC | 79.12 | CV-A11 | 100 | CV-A11 | 100 |
| S5_M954 | 528 | 2989 | NC_002058 | EVC | 81.52 | CV-A11 | 100 | CV-A11 | 100 |
| S6_M1295 | 594 | 2935 | AF363455 | EVG | 76.48 | NA | NA | NA | NA |
| S6_M2307 | 594 | 2935 | AF363455 | EVG | 76.48 | NA | NA | NA | NA |
| S6_M2449 | 594 | 2935 | AF363455 | EVG | 76.48 | NA | NA | NA | NA |
| S6_M2563 | 594 | 2935 | AF363455 | EVG | 76.43 | NA | NA | NA | NA |
| S6_M2688 | 594 | 2935 | AF363455 | EVG | 76.52 | NA | NA | NA | NA |
| S6_M2909 | 594 | 2935 | AF363455 | EVG | 76.56 | NA | NA | NA | NA |
| S6_M3314 | 594 | 2935 | AF363455 | EVG | 76.48 | NA | NA | NA | NA |
| S6_M3369 | 594 | 2935 | AF363455 | EVG | 76.48 | NA | NA | NA | NA |
| S6_M3408 | 594 | 2935 | AF363455 | EVG | 76.56 | NA | NA | NA | NA |
| S6_M350 | 594 | 2935 | AF363455 | EVG | 76.52 | NA | NA | NA | NA |
| S6_M3564 | 594 | 2935 | AF363455 | EVG | 76.52 | NA | NA | NA | NA |
| S6_M3674 | 594 | 2935 | AF363455 | EVG | 76.52 | NA | NA | NA | NA |
| S6_M3769 | 594 | 2935 | AF363455 | EVG | 76.56 | NA | NA | NA | NA |
| S6_M3786 | 594 | 2935 | AF363455 | EVG | 76.48 | NA | NA | NA | NA |
| S6_M3807 | 594 | 2935 | AF363455 | EVG | 76.48 | NA | NA | NA | NA |
| S6_M425 | 594 | 2935 | AF363455 | EVG | 76.57 | NA | NA | NA | NA |
| S6_M4329 | 594 | 2935 | AF363455 | EVG | 76.43 | NA | NA | NA | NA |
| S6_M4619 | 594 | 2935 | AF363455 | EVG | 76.43 | NA | NA | NA | NA |
| S6_M5011 | 594 | 2935 | AF363455 | EVG | 76.56 | NA | NA | NA | NA |
| S6_M5607 | 594 | 2935 | AF363455 | EVG | 76.52 | NA | NA | NA | NA |
| S6_M578 | 594 | 2935 | AF363455 | EVG | 76.60 | NA | NA | NA | NA |
| S6_M604 | 594 | 2935 | AF363455 | EVG | 76.48 | NA | NA | NA | NA |
| S6_M6231 | 594 | 2935 | AF363455 | EVG | 76.60 | NA | NA | NA | NA |
| S6_M7002 | 594 | 2935 | AF363455 | EVG | 76.52 | NA | NA | NA | NA |
| S6_M7040 | 594 | 2935 | AF363455 | EVG | 76.60 | NA | NA | NA | NA |
| S6_M7381 | 594 | 2935 | AF363455 | EVG | 76.52 | NA | NA | NA | NA |
| S6_M7464 | 594 | 2935 | AF363455 | EVG | 76.48 | NA | NA | NA | NA |
| S6_M752 | 594 | 2935 | AF363455 | EVG | 76.43 | NA | NA | NA | NA |
| S6_M7574 | 594 | 2935 | AF363455 | EVG | 76.56 | NA | NA | NA | NA |
| S6_M7790 | 594 | 2935 | AF363455 | EVG | 76.48 | NA | NA | NA | NA |
| S6_M837 | 594 | 2935 | AF363455 | EVG | 76.30 | NA | NA | NA | NA |
| S6_M8395 | 594 | 2935 | AF363455 | EVG | 76.52 | NA | NA | NA | NA |
| S6_M8545 | 594 | 2935 | AF363455 | EVG | 76.48 | NA | NA | NA | NA |
| S6_M8581 | 594 | 2935 | AF363455 | EVG | 76.39 | NA | NA | NA | NA |
| S6_M8663 | 594 | 2935 | AF363455 | EVG | 76.48 | NA | NA | NA | NA |
| S6_M8857 | 594 | 2935 | AF363455 | EVG | 76.43 | NA | NA | NA | NA |
| S6_M989 | 594 | 2935 | AF363455 | EVG | 76.60 | NA | NA | NA | NA |
| S7_M4604 | 594 | 2935 | AF363455 | EVG | 78.38 | NA | NA | NA | NA |
| S7_M511 | 594 | 2935 | AF363455 | EVG | 78.42 | NA | NA | NA | NA |
| S7_M6946 | 594 | 2935 | AF363455 | EVG | 78.46 | NA | NA | NA | NA |
| S7_M7806 | 594 | 2935 | AF363455 | EVG | 78.38 | NA | NA | NA | NA |
| S8_M3182 | 594 | 2932 | AF363455 | EVG | 71.87 | NA | NA | NA | NA |
| S8_M6960 | 594 | 2932 | AF363455 | EVG | 71.74 | NA | NA | NA | NA |
| S8_M9221 | 594 | 2932 | AF363455 | EVG | 71.74 | NA | NA | NA | NA |

**Table S4.** Top two BLASTn hits of representatives of each EV type recovered from sludge in Kentucky, USA

|  |  |  | Top 2 BLASTn Hits | | | |
| --- | --- | --- | --- | --- | --- | --- |
| **Amplicon-Unique-ID** | **BLAST**  **result** | **Type** | **Accession number** | **% Identity** | **Accession number** | **% Identity** |
| S3_M1087 | EVG | G15 | MN734577.1 | 78.80 | KT265894.2 | 76.76 |
| S3_M6117 | EVG | G15 | MN734577.1 | 78.71 | KT265894.2 | 76.88 |
| S3_M1933 | EVC | CV-A24 | MW373950.1 | 80.74 | EF015036.1 | 79.77 |
| S3_M2103 | EVC | CV-A24 | MW373950.1 | 80.66 | EF015036.1 | 79.69 |
| S3_M3761 | EVA | CV-A2 | MT641397.1 | 96.86 | KP289361.1 | 85.47 |
| S3_M4055 | EVA | CV-A2 | MT641397.1 | 96.73 | KP289361.1 | 85.35 |
| S3_M5429 | CanPV | NA | KU871312.1 | 84.84 | MW118112.1 | 84.53 |
| S3_M5434 | CanPV | NA | KU871312.1 | 84.80 | MW118112.1 | 84.48 |
| S5_M248 | EVC | CV-A11 | JF260918.1 | 80.95 | JF260919.1 | 80.92 |
| S5_M307 | EVC | CV-A11 | AF465512.1 | 81.38 | AF499638.1 | 81.26 |
| S6_M989 | EVG | G1 | LC316790.1 | 78.93 | MZ679264.1 | 78.43 |
| S6_M2909 | EVG | G1 | LC316790.1 | 78.97 | MZ679264.1 | 78.41 |
| S7_M6946 | EVG | G2 | MW504538.1 | 82.38 | LC316793.1 | 79.05 |
| S7_M7806 | EVG | G2 | MW504538.1 | 82.27 | LC316793.1 | 78.92 |
| S8_M3182 | EVG | G9 | KT265894.2 | 83.70 | KT265961.2 | 83.40 |
| S8_M6960 | EVG | G9 | KT265894.2 | 83.65 | KT265961.2 | 83.36 |
